# Micro-Doppler measurement of insect wing-beat frequencies with W-band coherent radar

**DOI:** 10.1038/s41598-017-01616-4

**Published:** 2017-05-03

**Authors:** Rui Wang, Cheng Hu, Xiaowei Fu, Teng Long, Tao Zeng

**Affiliations:** 1 School of Information and Electronics, Beijing Institute of Technology, Beijing, 100081 China; 2 Beijing Key Laboratory of Embedded Real-time, Information Processing Technology, Beijing, 100081 China; 3 State Key Laboratory for Biology of Plant Diseases and Insect Pests, Institute of Plant Protection, Chinese Academe of Agricultural Sciences, Beijing, 100193 China; 4 Department of Electronic Engineering, Tsinghua University, Beijing, 100084 China

## Abstract

The wingbeat frequency of insect migrant is regarded potentially valuable for species identification and has long drawn widespread attention in radar entomology. Principally, the radar echo signal can be used to extract wingbeat information, because both the signal amplitude and phase could be modulated by wing-beating. With respect to existing entomological radars, signal amplitude modulation has been used for wingbeat frequency measurement of large insects for many years, but the wingbeat frequency measurement of small insects remains a challenge. In our research, W-band and S-band coherent radars are used to measure the insect wingbeat frequency. The results show that the wingbeat-induced amplitude modulation of W-band radar is more intense than that of the S-band radar and the W-band radar could measure the wingbeat frequency of smaller insects. In addition, it is validated for the first time that the signal phase could also be used to measure the insect wingbeat frequency based on micro-Doppler effect. However, whether the wingbeat frequency measurement is based on the amplitude or phase modulation, it is found that the W-band coherent radar has better performance on both the measurement precision and the measurable minimum size of the insect.

## Introduction

Insects, with the most speciose and abundant biodiversity, play an important role in many phenomena, e.g. pollination, biological control, physical decomposition and providing a wide range of products^1^. Insects are the only invertebrates equipped with wings and capable of powered flapping flight, which they use to conduct essential activities such as seeking refuges, locating host plants, avoiding predators, finding mates, searching for oviposition places and long distance migration. Many more than billions of insects migrate annually, which provides major ecosystem services, causes serious crop damage, and spreads diseases of plants, humans and their livestock^2,3^.

Because most species of migratory insect are nocturnal, fly hundreds of meters above the ground, and are too small for movement observation and individual tracking, the knowledge of insect migration lags somewhat behind that of vertebrates, such as birds, bats, and terrestrial and marine mammals. However, radar provides a means of directly detecting insects migrating in the lower atmosphere without perturbing them. Special-purpose entomological radars are able to detect intense insect migration and to determine the characteristics of each individual (such as its flying altitude, displacement speed, heading direction, body alignment and body size)^2,4^. With the flight parameters above detected by radar, new discoveries of the ecological and biological behaviors of migration insects are constantly published. For example, the phenomena of the large-scale taking-off, high-density layering, and common orientation of high-flying migrants were first directly observed by entomological radars^2^. Overall, entomological radars have significantly extended the knowledge of insect migration.

Although important progress has been made by previous radar studies, target identification has long been a challenge in radar entomology. Aerial net sampling carried out by planes, kytoons, or kites can provide direct evidences for the identity of windborne migration insects^5,6^. Searchlight trapping is also a direct aid in identifying large insects migrating at night^7^. However, the direct sampling of aerial targets is a labor-intensive and time-consuming work, and is not practicable for the real-time monitoring of windborne migratory insects. The identification of flying targets detected by entomological radar provides an effective solution to this seemingly difficult problem.

The species identification of migratory insects based on entomological radar is a quite complex procedure. It has often been suggested that information extracted from the radar echo (such as the radar cross-section, body mass, and wingbeat frequency.) could be used, at least to some extent, to identify targets. Wingbeat frequency has been regarded as potentially valuable for target identification^4,8–10^, and in some situations, radar measurements of the wingbeat frequency provide a very effective means of identifying airborne insects. Species with longer wings tend, in general, to have lower wingbeat frequencies^11^. The wingbeat frequency could be detected based on signal amplitude modulation by both the early scanning radars^4^ in non-scanning mode and the more recent zenith-pointing linear-polarized small-angle conical-scan (ZLC) radars^8–10,12^. The success rate of the wingbeat frequency retrieval for large targets is approximately 46%^10^, but for small insects, the wingbeat frequency measurement is difficult. This is probably because the amplitude modulation of the echo signal from the wing-beating is too weak to be detected for smaller insects.

In the existing entomological radars, a non-coherent system design is adopted to detect the returned signal amplitude of insects. With the rapid development of radar techniques, coherent radars have been widely applied. Both the signal amplitude and carrier phase could be utilized, and it allows extracting the Doppler frequency induced by the moving target, which is defined as a frequency shift of the received signal relative to the transmitted signal^13^. If the structural target also contains vibrational or rotational components, it may induce frequency modulation and generate sidebands about the Doppler frequency shift. The modulation due to vibrations is called the micro-Doppler effect^14^. The micro-Doppler effect of bird wing-flapping has been investigated^15^ and used to measure bird wing-flapping pattern for target classification^16,17^.

In our research, W-band and S-band coherent radars were used to measure the insect wingbeat frequency. Through signal amplitude and phase analysis, we examined the wingbeat frequency measurement capability for W-band and S-band coherent radars. It was validated for the first time that the signal phase could also be used to measure the wingbeat frequency of insects based on the micro-Doppler effect, and the precision was further evaluated using a stroboscope. In addition, a comparison of wingbeat frequency measurements between W-band and S-band radars was also performed, to show which band radar is more suitable to measure the wingbeat frequency of insects.

## Methods

### Experimental equipment and configuration

**Figure 1:**
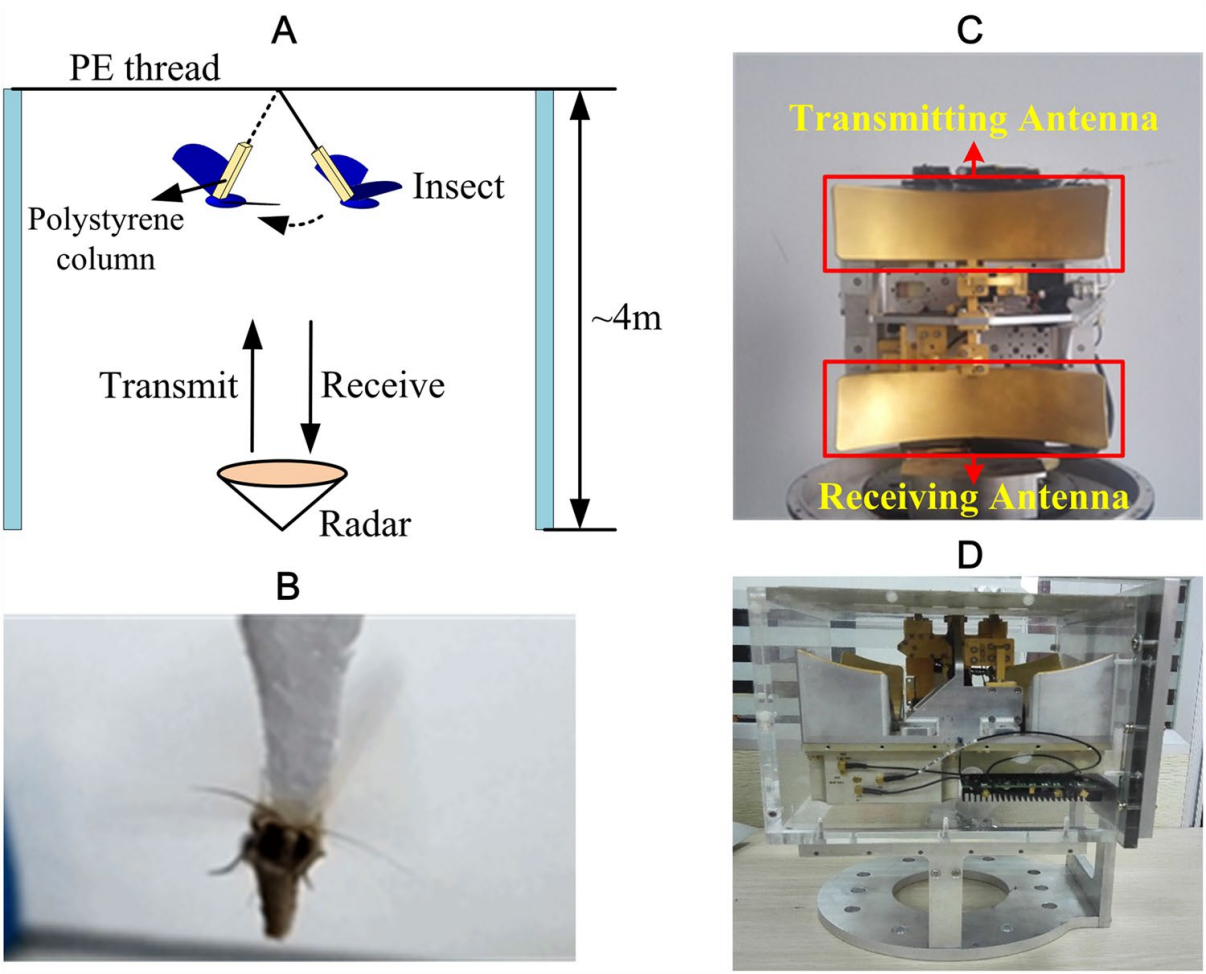
(**A**) The experimental geometry of wingbeat frequency measurement; (**B**) The suspended *Mythimna separata*; (**C**) The W-band coherent radar (top view); (**D**) The W-band radar (side view). Note: The radar system has dual-antenna design for transmitting and receiving to solve the radar blind distance problem. These two antennas are corresponding to the top and bottom golden components as marked in Fig. 1(C).

The wingbeat frequency measurement experiments were carried out using a W-band coherent radar (Fig. 1(C,D)) with a wavelength of 3.2 mm and an S-band coherent radar with a wavelength of 90 mm. The experimental geometry is shown in Fig. 1(A). A piece of low-scattering polystyrene foam is adhered to insect back (Fig. 1(B)). Then a thread goes through the top part of this polystyrene column by a needle and finally it is tethered to a PE thread that is not rigid as shown in Fig. 1(A), where the PE thread is an ultra-high molecular weight polyethylene fiber composite line with a line width of only 0.1 mm in order to prevent the ambient clutter from interfering with the insect echo. Experimental results show that the polystyrene foam and PE thread only contributed a small portion of the radar echo, which has no effect on wingbeat frequency measurement. The radar beam faces vertically upward and the insect echo can be acquired while the insect flies within the radar beam. It should be mentioned that this is the same target aspect as that used in many current vertical-beam entomological radars^10,11^. In addition, the ability to detect wing-beat modulation can be expected to be aspect-dependent both with the existing methods and this new method. Thus, in our experiment, the polystyrene foam is also used to limit the flying attitude of the insect, so that the insect abdomen could be toward the radar and the direction of wing-beating could be as much parallel with the radar light of sight as possible.

For the W-band radar, a frequency modulated continuous wave (FMCW) with a sweep time of 0.5 ms was adopted as the transmitted signal waveform. The total signal bandwidth is 1.2 GHz, so that the range resolution is 0.125 m. In contrast, for the S-band radar, a stepped-frequency pulse-train (SFPT) signal was adopted as the transmitted signal waveform with a synthetic bandwidth of 320 MHz, providing a range resolution of 0.47 m. The high-range resolution brought by the wide bandwidth is helpful in suppressing clutter and enhancing the radar detection performance for weak targets.

### Micro-Doppler analysis and valid data selection

**Figure 2:**
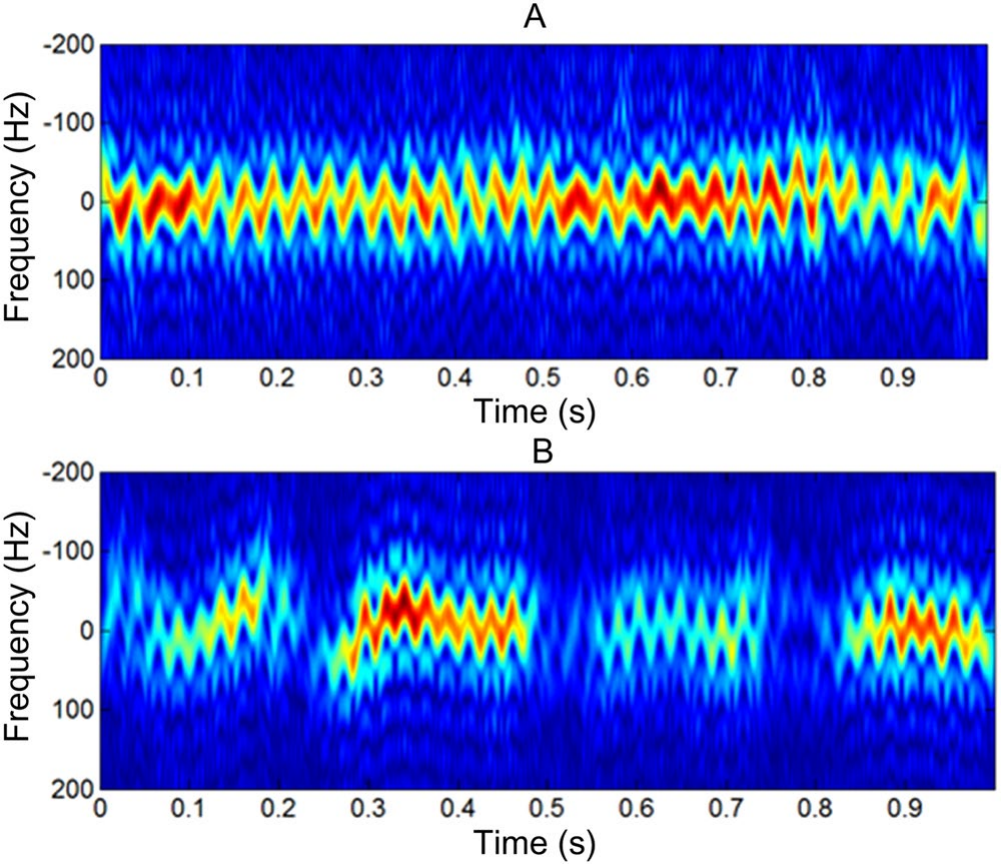
Examples of the time-frequency analysis by STFT: Time-frequency spectrograms of (**A**) *Agrotis ypsilon* and (**B**) *Mamestra brassicae*. The sliding window length is 200 ms with the frequency resolution of 5 Hz.

We found that during the radar observation, the tethered insect may fly out of the radar beam, and their wings also do not flap all the time. Thus, a selection of valid data for the wingbeat measurement is required. As a useful micro-Doppler analysis tool, the short-time Fourier transform (STFT) is used in our signal processing at the initial stage to evaluate whether the received signal has micro-Doppler modulation by wing-beating (Fig. 2). It can be seen that the STFT gives a good indication of the signal’s frequency variation with time. In comparison with the signal in Fig. 2(A), which has a wingbeat modulation for the given period of 1 s, the signal in Fig. 2(B) has a few interruptions in its frequency modulation. Therefore, the valid data segments can be selected for further wingbeat frequency extraction. Please see the supplementary materials for details.

Note that the experimental insects include *Athetis lepigone*, *Agrotis ypsilon*, *Agrius convolvuli*, which were obtained by searchlight trapping. The insects with body lengths between 10 mm to 39 mm were subjected to W-band radar measurement while those with body lengths from 13 mm to 42 mm were measured by S-band radar. It can be seen that the small insects were evaluated in both W-band and S-band radar measurements. However, through the micro-Doppler analysis, it is found that valid wingbeat-induced data were not acquired for all insects even if the wing-beating occurred. Thus, only the insects with valid data were subjected to the wingbeat-induced spectrum analysis and wingbeat frequency extraction, as described in the Results section (see Tables 1 and 2).

**Table 1:** Signal power statistics of different spectral components.

Radar	No.	Species	Average power of spectral component <10 Hz	Average power of spectral component >10 Hz	Power Ratio
W-band radar	1	*Diaphania perspectails*	6.4 × 10^−4^	4.8 × 10^−5^	7.5%
	2	*Krananda latimarginaria*	1.8 × 10^−3^	1.5 × 10^−4^	8.3%
	3	*Agrotis ypsilon*	1.9 × 10^−3^	1.3 × 10^−4^	6.8%
	4	*Polia illoba*	1.9 × 10^−3^	3.6 × 10^−4^	18.9%
	5	*Mythimna separata*	1.8 × 10^−3^	8.3 × 10^−5^	4.6%
	6	*Plusia agnata*	1.6 × 10^−3^	1.3 × 10^−4^	8.1%
	7	*Athetis lepigone*	7.3 × 10^−4^	3.0 × 10^−5^	4.1%
	8	*Odonestis pruni*	2.9 × 10^−3^	1.8 × 10^−4^	6.2%
	9	*Adristyrannus sp*.	1.8 × 10^−3^	1.3 × 10^−4^	7.2%
	10	*Pergesa elpenorlewisi*	1.5 × 10^−4^	6.2 × 10^−6^	4.1%
	11	*Mamestra brassicae*	1.3 × 10^−2^	2.9 × 10^−4^	2.2%
	12	*Macroglossum corythus luteata*	4.2 × 10^−2^	5.4 × 10^−4^	1.3%
	13	*Agrotis tokionis*	1.2 × 10^−1^	8.3 × 10^−3^	6.9%
S-band Radar	14	Unknown geometrid moth	7.4 × 10^−2^	5.0 × 10^−3^	6.8%
	15	*Clanis bilineata*	9.4	6.1 × 10^−2^	0.6%
	16	*Agrotis ypsilon*	8.1 × 10^−2^	3.0 × 10^−3^	3.7%
	17	*Agrius convolvuli*	4.7	1.0 × 10^−2^	0.2%
	18	*Teretra japonica*	9.5 × 10^−1^	7.0 × 10^−3^	0.7%
	19	Unknown moth	7.6 × 10^−1^	5.0 × 10^−3^	0.6%
	20	*Macroglossum corythus luteata*	1.2 × 10^−1^	4.0 × 10^−3^	3.3%

Note: The “Power Ratio” in last column represents signal power ratio of spectral component lower than 10 Hz to spectral component higher than 10 Hz. In addition, note that W-band and S-band radars have different transmitted power and system gain. As no radar calibration is made, the given signal powers in the first two columns only represent relative quantized sample value, rather than the absolute backscattered signal intensities of insects. All the measured insects are moths (Lepidoptera).

**Table 2:** The wingbeat frequency measurement results using W-band and S-band radars.

Radar	No.	Body (mm)		Wingspan/mm	Wingbeat Freq. (Hz)		
		Length	Width		Amplitude method	Phase method	Stroboscope
W-band radar	1	21	4	36	29.00	28.88	28.57
	2	20	4	36	29.13	29.13	29.00
	3	25	5	40	33.27	30.63	30.44
	4	18	4	37	32.00	32.25	32.28
	5	20	5	39	34.75	34.38	34.07
	6	16	4	32	37.25	37.38	37.47
	7	10	3	20	37.75	37.50	37.25
	8	23	5	44	36.63	37.75	37.00
	9	23	6	42	39.75	39.88	39.00
	10	39	10	58	40.13	40.13	40.18
	11	18	4	34	43.50	42.75	42.00
	12	31	8	50	49.75	50.25	49.61
	13	24	5	42	56.13	56.38	56.33
S-band Radar	14	27	6	63	21.97	19.53	20.24
	15	41	10	102	24.41	24.41	25.41
	16	24	5	42	29.30	30.52	30.65
	17	42	10	82	32.96	31.74	32.98
	18	35	7	70	35.16	35.16	36.70
	19	30	6	44	41.50	41.50	40.34
	20	30	7	47	63.48	63.71	62.75

Note: These experimental results are arranged in order of wingbeat frequency with the lowest at top. In addition, because the insect is limited and adhered to a piece of polystyrene foam in the experiment, the wing beating may be affected and the typical value of wingbeat frequency of these species cannot be used as the reference frequency for comparisons.

## Results

### Spectrum component analysis for W-band and S-band radars

The echo signal of the insects is first analyzed to assess the sensitivity of the amplitude modulation to wing-beating for both the W-band and S-band radars. The signal amplitude modulation is mainly composed of two components, one a low frequency component induced by the body movement which mainly reflects the target aspect and relative position changes relative to the radar and the other a high frequency component induced by wing-beating. In general, if the radar cross-section of the insects is of concern, we only need consider the intensity of the low-frequency component. When the wingbeat frequency measurement is required, the intensity of the high frequency component will be our main consideration. Obviously, to achieve better measurement of wingbeat frequency, we hope that the average power ratio of the high-frequency component to the low-frequency component is high. With respect to insects with body lengths ranging from 10 mm–42 mm in our experiment, the scattering mechanism is different, as Rayleigh scattering mainly occurs in the S-band and resonance or optical scattering in the W-band. Thus, this average power ratio could be quite different. Next, a spectral component analysis is performed using experimental data at the W-band and S-band, respectively.

**Figure 3:**
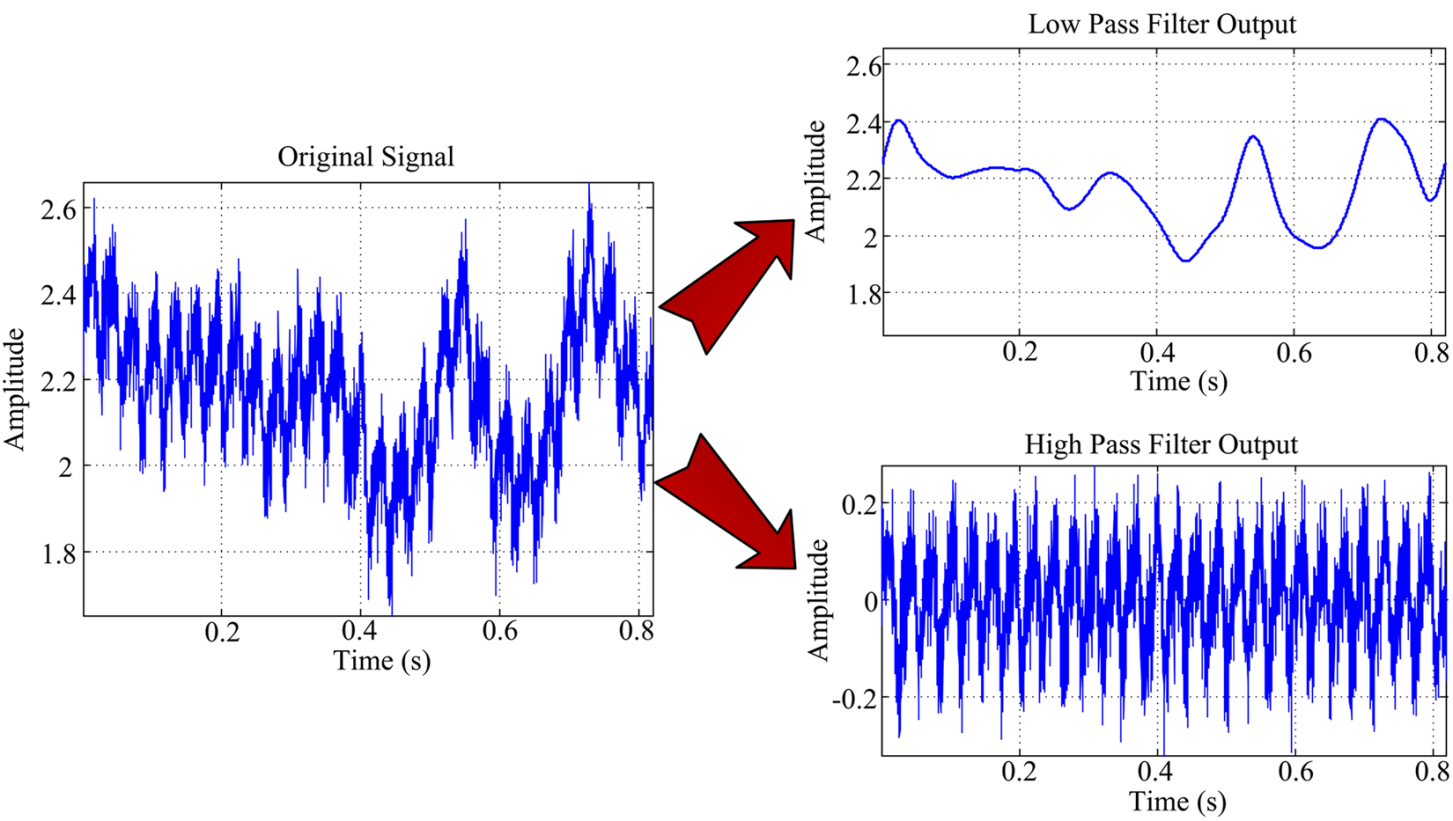
The separation of the body-movement and wing-beating signal modulations.

Since the backscattering signal of the insect is linearly received, the received signal power can be directly used to analyze the backscattering capability of the insect. According to the analysis above, the signal variation induced by the insect’s turning and translational movement is slower than that by wing-beating. The wingbeat frequency of the insect is generally higher than 10 Hz, so the wingbeat-modulated signal can be separated using high-pass filtering with a cutoff frequency of 10 Hz (Fig. 3). Finally, the statistics of the signal power induced by the slow body movement and quick wingbeat can be obtained, and the average signal power ratios of these two spectral components are shown in Table 1. For various insects, these power ratios are different, probably depending on the insect biological structure. However, it is noteworthy that the average power ratios in the W-band are one order of magnitude higher than those in the S-band in general. This implies that the wingbeat-induced signal intensity is relatively weaker in the S-band, which could deteriorate the wingbeat measurement capability. This could also be the reason why the S-band radar is only able to measure the wingbeat frequency of insects with a body length larger than 24 mm (see Table 2). For smaller insects, the amplitude modulation by the insect’s turning and translational movement can be detected, but the wingbeat frequency measurements still fail in our S-band experiments.

### Wingbeat frequency measurement and validation

**Figure 4:**
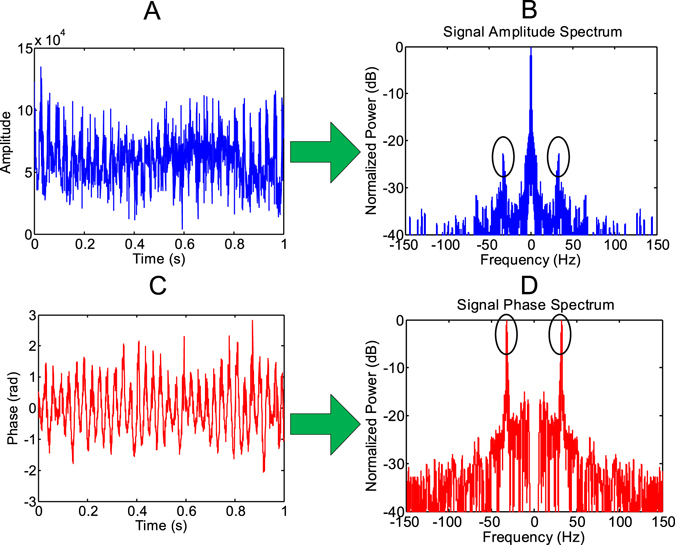
Wingbeat frequency extractions. The presented results are corresponding to *Agrotis ypsilon* (No.3) in W-band radar experiment. Both the extracted amplitude and phase modulations are shown in (**A**) and (**C**). Then Fourier transform is applied to measure wingbeat frequency as shown in (**B**) and (**D**), respectively. The black ellipses are used to mark the peaks that correspond to wingbeat frequencies.

With respect to the wingbeat-induced signal, both the amplitude and phase can be extracted for coherent radars. The amplitude modulation can be used to measure the wingbeat frequency by Fourier spectral analysis (Fig. 4(A)), which has been applied in current entomological radars^9^. In our research, it is first validated that the wingbeat-induced signal phase, which can be extracted from the high frequency signal component after a frequency-domain filter, could also be used to measure the wingbeat frequency based on the micro-Doppler effect (Fig. 4(B)). For the specific signal processing method, please refer to the supplementary materials. Meanwhile, to fully evaluate the accuracy of the radar measurement, a stroboscope is also used to provide a referenced wingbeat frequency. The final results of the wingbeat frequency retrieval are shown Table 2.

Next, we performed a statistical analysis of the wingbeat frequency measurements of the W-band and S-band radars (see Table 3). For the amplitude-based method, the standard deviation (SD) is 0.89 Hz for the W-band radar and 1.29 Hz for the S-band radar, while for the phase-based method, the SD is 0.33 Hz for the W-band radar and 1.06 Hz for the S-band radar. It can be found that the signal phase shows a higher precision in the wingbeat frequency measurement than the signal amplitude, although the precision difference is not quite significant especially in the S-band. These results prove that the micro-Doppler phase variation is consistent with the insect wing-beating, and it could indeed be used to measure the wingbeat frequency. In addition, the statistical results show that the W-band coherent radar achieves higher measurement precision than the S-band coherent radar, no matter whether the signal amplitude or phase is utilized. For the amplitude-based method, according to the signal intensity analysis above, the amplitude modulation by wing-beating in the W-band is more intense; hence, it is supposed to have a higher SNR and measurement precision. For the phase-based method, because the wingbeat-induced signal phase could be extracted more accurately under a higher SNR and the phase is principally more sensitive to micro-vibration at a shorter wavelength, it should have better measurement in the W-band. Therefore, it is reasonable that the W-band coherent radar is able to achieve higher measurement precision. Moreover, note that the S-band radar is only able to measure the wingbeat frequency of larger insects with a body length of more than 24 mm in our experiments, while the W-band could measure the wingbeat frequency of *Athetis lepigone*, with a body length of 10 mm. Overall, this suggests that the W-band coherent radar is a favorable selection for wingbeat frequency measurements.

**Table 3:** Standard deviation of wingbeat frequency measurement errors for W-band and S-band radars.

Statistical measure	Amplitude-based method		Phase-based method	
	W-band radar	S-band radar	W-band radar	S-band radar
Standard deviation (Hz)	0.89	1.29	0.33	1.06

Note: These statistics of the differences between the stroboscope and radar measurement values are with one datum for each specimen (n = 13 for W-band and 7 for S-band).

## Discussion

The experiment results show that the micro-Doppler phase could be used to measure the wingbeat frequency and the precision is higher than that of the amplitude-based method. However, it can be seen that the precision difference is not quite significant, especially for the S-band. Through Cramer-Rao lower bound (CRLB) analysis, for the amplitude-based method, the minimal wingbeat frequency estimation error is affected by the sampling rate, sampling number and SNR, while for the phase-based method, the effective vibration amplitude and radar wavelength are also involved. From the extracted micro-Doppler phase in our experiment, we found that the effective vibration amplitude induced by wing-beating was on the millimeter order of magnitude or even smaller. Considering all these factors, the minimal wingbeat frequency estimation error based on phase modulation is at least three times lower than that based on amplitude modulation. However, it can be seen that the precision difference between the amplitude-based and phase-based methods is not consistent with the theoretical analysis. A possible reason is that our signal processing method is not a minimum-variance unbiased estimator. Therefore, the optimal estimation method still needs to be investigated further.

It is widely acknowledged that the sensitivity of the micro-Doppler phase to vibration is inversely proportional to the radar wavelength. Thus, it would seem that the wingbeat frequency measurement precision based on phase modulation in the W-band should be thirty times higher than that in the S-band. However, through CRLB analysis, the relationship between the wingbeat frequency estimation error and radar wavelength is shown to not be simply linear (see eq. 13 in supplementary materials). For the effective vibration amplitude ranging from 0.1 mm to 1 mm, the precision benefit from the W-band is approximately 1.1 to 3.7 times relative to the S-band for the wingbeat frequency measurement.

By applying a band-pass filter around the frequency of wing-beating on the signal phase, the phase modulation by wing-beating can be extracted, and hence the effective vibration amplitude can be analyzed. As mentioned above, we found that the effective vibration amplitude was on the millimeter order of magnitude or even smaller. However, as we know, the actual wing-beating amplitude is usually larger than 1 mm or even 10 mm, at least for larger insects. This implies that the signal echo induced by wing-beating may be not only from the insect wings, but also from other parts of the insect, probably such as changes in body shape or attitude by wing beating. However, the relationship between the effective vibration and the real vibration induced by wing-beating is still unclear, as it involves a complex scattering mechanism and biological structure. Therefore, it needs further investigation in the future.

The species identification of migratory insects based on entomological radar is a very complex procedure. It is necessary to thoroughly consider the exterior biological parameters of the insect itself, e.g. wingbeat frequency, body size, body shape, and body mass. This paper mainly focused on developing a precise wingbeat frequency measurement. In the published literature, the body mass of insects has been widely studied based on RCS, whereas the body size and body shape retrievals are still unsolved, although some qualitative analysis has been investigated. The precise measurements of these biological parameters will be one of our most important research tasks in the future. In addition, note that the wingbeat frequency of insect migrants could be affected by the ambient environment (temperature, humidity, etc.) and biological factors (sex, age, etc.). While the wingbeat frequency is used to support insect identification, its variations with environmental and biological factors must be taken into consideration. Therefore, the relationship between the wingbeat frequency and these factors is also essential to be fully investigated in the future.

In summary, we made the following two improvements in this study compared with the former researches on insect wingbeat frequency measurement. The first one is to apply the micro-Doppler technique to the insect wingbeat frequency measurement. The experimental results showed that the echo signal phase of the insect can also be used to extract the wingbeat frequency, and good measurement precision can be achieved. Second, an experimental comparison between the W-band and S-band radars revealed the advantages of using shorter wavelengths to measure the insect wingbeat frequency. Whether using traditional the amplitude-based or new phase-based methods, the W-band coherent radar showed better performance on both the measurement precision and the measurable minimum size of the insect.

## Electronic supplementary material

Supplementary Materials for Micro-Doppler measurement of insect wing-beat frequencies with W-band coherent radar
