# Author Correction: Micro-Doppler measurement of insect wing-beat frequencies with W-band coherent radar

**DOI:** 10.1038/s41598-018-21310-3

**Published:** 2018-03-01

**Authors:** Rui Wang, Cheng Hu, Xiaowei Fu, Teng Long, Tao Zeng

**Affiliations:** 1 School of Information and Electronics, Beijing Institute of Technology, Beijing, 100081 China; 2 Beijing Key Laboratory of Embedded Real-time Information Processing Technology, Beijing, 100081 China; 3 State Key Laboratory for Biology of Plant Diseases and Insect Pests, Institute of Plant Protection, Chinese Academe of Agricultural Sciences, Beijing, 100193 China; 4 Department of Electronic Engineering, Tsinghua University, Beijing, 100084 China

Correction to: *Scientific Reports* 10.1038/s41598-017-01616-4, published online 03 May 2017

The Acknowledgements section in this Article was omitted. The Acknowledgements section should read:

“This work was supported in part by the National Natural Science Foundation of China (Grant 61427802) and in part by the Science and Technology Innovation Plan of Beijing Institute of Technology (Grant 2016CX01002).”

