# Supplementary material for: Micro-Doppler measurement of insect wing-beat frequencies with W-band coherent radar
Source: Sci Rep. 2017 May 3;7:1396. doi: 10.1038/s41598-017-01616-4 (PMC5431090; doi:10.1038/s41598-017-01616-4)
Supplement: Supplementary file 1 — Supplementary Materials for Micro-Doppler measurement of insect wing-beat frequencies with W-band coherent radar [file 41598_2017_1616_MOESM1_ESM.pdf]

Supplementary Materials for

Micro-Doppler measurement of insect wing-beat frequencies with W-band coherent radar

Rui Wang, Cheng Hu*, Xiaowei Fu, Teng Long, Tao Zeng

*Corresponding author. Email: hucheng.bit@gmail.com

This PDF file includes

Signal processing method
Precision analysis of wingbeat frequency measurement
Figs. S1 to S3
References

Signal processing method

Signal processing of high resolution range profile. The resolution of range profile determines the distinguishability of two targets along the range direction. The high resolution is beneficial to separate the effective target from the ambient clutter. In our experiments, the frequency modulated continuous wave (FMCW) and stepped-frequency pulse-train (SFPT) waveforms are adopted as the transmitted signal in W-band radar and S-band radar, respectively.

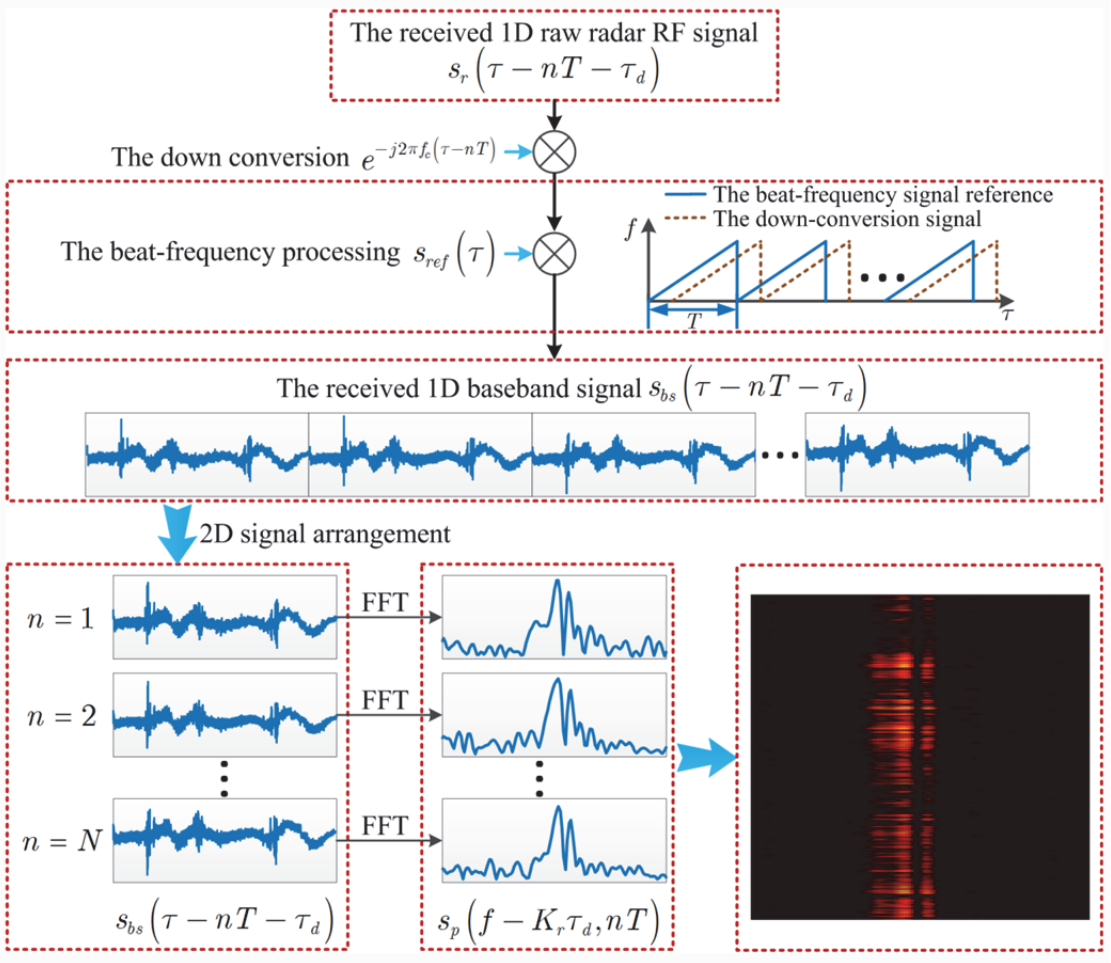

Figure S1. The block diagram of signal processing from the raw received RF radar signal to range compression image.

For a point target, taking FMCW as an example, the block diagram of signal processing of high resolution range profile is shown in Fig. S1. At the beginning, the original received radio frequency (RF) signal can be expressed as

$$s_r\left(\tau - nT - \tau_d\right) = \sigma\left(\tau\right)\mathrm{rect}\left\{\frac{\tau - nT - \tau_d}{T}\right\}e^{j2\pi f_c\left(\tau - nT - \tau_d\right) + j\pi K_r\left(\tau - nT - \tau_d\right)^2} \quad (1)$$

where τ represents the time variable. The K_r is the frequency sweep rate. The backscattered signal of the target is denoted as $\sigma\left(\tau\right)$. The echo delay of target is denoted as $\tau_d = 2R\left(\tau\right)/c$, where $R\left(\tau\right)$ stands for the range between the radar and the target, and c is the light speed. In addition, $\mathrm{rect}\{\cdot\}$ represents the rectangular function with the width of T which can be considered as the signal repeated period, and n is the index of rectangular function.

First of all, the down conversion is performed by multiplying $e^{-j2\pi f_c\left(\tau - nT\right)}$ to convert the raw echo into the baseband signal, which can written as

$$s_{bs}\left(\tau - nT - \tau_d\right) = \sigma\left(\tau\right)e^{j\phi\left(\tau\right)}\mathrm{rect}\left\{\frac{\tau - nT - \tau_d}{T}\right\}e^{j\pi K_r\left(\tau - nT - \tau_d\right)^2} \quad (2)$$

where $\phi\left(\tau\right) = -2\pi f_c\tau_d = -4\pi R\left(\tau\right)/\lambda$ represents the carrier phase of the target. It can be found that the range displacement of 1.5 mm can lead to phase variation of 180 degree for W-band. Therefore, the carrier phase is quite sensitive to micro-vibration, especially for insect body flexing or vibrating caused by wingbeat.

Next, the beat-frequency processing between the baseband signal and the signal reference is implemented, where the signal reference is selected as the transmitted signal in general. Thus, after simplification, the output signal can be written as

$$s_{bs}\left(\tau - nT - \tau_d\right) \approx \sigma\left(\tau\right)e^{j\phi\left(\tau\right)}\,\mathrm{rect}\left\{\frac{\tau - nT - \tau_d}{T}\right\}e^{-j2\pi K_r\tau_d\left(\tau - nT\right)} \tag{3}$$

where $\pi K_r \tau_d^2$ is ignored because it far smaller than $\phi\left(\tau\right)$.

Then, the beat-frequency signal is arranged to 2D signal format according to signal repeated period index. Fast Fourier transformation (FFT) is applied on each individual signal to achieve the high resolution range compression. Finally, the high-resolution range profile can be expressed as

$$s_r\left(f - K_r\tau_d, nT\right) \approx \sigma\left(nT\right)e^{j\phi\left(nT\right)}\,\mathrm{sinc}\left\{\pi K_r T\left(f - K_r\tau_d\right)\right\} \tag{4}$$

where $\mathrm{sinc}\{\cdot\}$ is the sinc function and its resolution is determined by signal bandwidth of $K_r T$, f is the transmitting frequency. In addition, note that the signal repeated period T is usually in microsecond order of magnitude, which is 0.5 ms for our W-band radar. As thus, $\sigma\left(\tau\right)$ and $\phi\left(\tau\right)$ could be considered non-variational during signal repeated period and is simplified as $\sigma\left(nT\right)$ and $\phi\left(nT\right)$.

With respect to SFPT, the whole block diagram from the raw received radar signal is the same and the only difference is the range compression processing. The signal processing of range compression in detail refers to ref [1].

Target detection based on wingbeat frequency measurement. As the suspended insect is mostly in flat flight, the direction of which is perpendicular to the radar line of sight, the returned signal from insect after range compression could not migrate through a range resolution cell during radar observation interval. Thus, the signal integration can be directly done along the same range resolution cell without range migration correction, and subsequently the target detection was performed to identify the effective range cell (Fig.

S2(a)). Owing to the clutter in the experimental environment, two targets were detected in the same range profile. As the insect has wingbeat behavior while the clutter target does not, it is possible to identify the insect target from the signal spectrum of amplitude or phase. In our experiment, the signal carrier phase is extracted to measure the wingbeat frequency as shown in Fig. S2(b). Based on signal phase spectrum analysis, the insect target is able to be identified according to its wingbeat frequency.

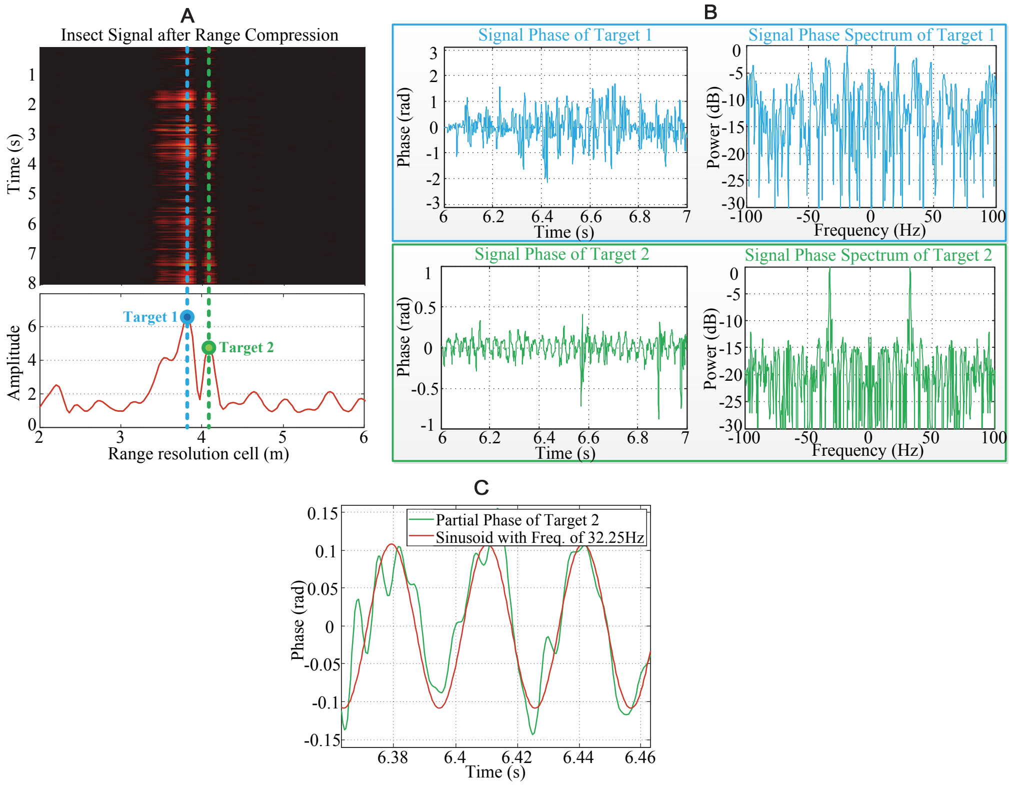

Figure S2. (a) Insect signal detection after range compression; (b) The wingbeat frequency extraction utilizing the signal carrier phase along the range cell of the detected targets; (c) The partial phase of Target 2 versus the fitted sinusoid curve with the measured wingbeat frequency of 32.25Hz. Note: The clutter is existent in the experimental environment. Fortunately, the high resolution range profile can distinguish the clutter and insect target. Target 1 is the clutter signal rather than the insect and hence the wingbeat frequency cannot be observed in the phase spectrum. Target 2 is the insect of *Polia illoba.* The frequency of 32.25Hz can be observed in the phase spectrum.

Wingbeat frequency measurement based on micro-Doppler phase. From the high resolution range profile, the range cell of target can detected (Fig. S2(a)) and then the amplitude $\sigma\left(nT\right)$ and carrier phase $\phi\left(nT\right)$ of backscattered signal of the target can be extracted. Without loss of generality, in the range cell of insect target, we assume that there are three components in the returned signal from the clutter, the translational movement of insect body and the micro-vibration caused by wing-beating, respectively. Thus, the signal along the range cell can be formulated as

$$\sigma\left(t\right)e^{j\phi\left(t\right)} = s_{clutter} + \sigma_{body}\left(t\right)e^{j\phi_b\left(t\right)} + \sigma_{wingbeat}\left(t\right)e^{j\phi_w\left(t\right)} \tag{5}$$

where nT is substituted by t for state convenience later, $\phi_b\left(t\right)$ is the Doppler phase induced by translational movement of insect body and $\phi_w\left(t\right)$ is the micro-Doppler phase modulation by wing-beating.

Next, the wing-beating is considered as a harmonic motion, and thus the returned signal amplitude and phase is modulated periodically as

$$\sigma_{wingbeat}\left(t\right) = \sigma_0 \sin\left(2\pi f_w t + \varphi_0\right); \quad \phi_w\left(t\right) = \phi_b\left(t\right) + \frac{4\pi A}{\lambda}\sin\left(2\pi f_w t\right) \tag{6}$$

In (6), A is the effective vibration amplitude induced by wing-beating and f_w represents wingbeat frequency.

Utilizing Bessel series, the wingbeat component in (3) can be expressed as

$$\begin{aligned} \sigma_{wingbeat}\left(t\right)e^{j\phi_w\left(t\right)} = &-j\frac{\sigma_0}{2}\sum_{n=-\infty}^{\infty} J_n\left(4\pi A/\lambda\right)e^{j2\pi\left(nf_w+f_w\right)t+j\phi_b\left(t\right)+j\varphi_0} \\ &+ j\frac{\sigma_0}{2}\sum_{n=-\infty}^{\infty} J_n\left(4\pi A/\lambda\right)e^{j2\pi\left(nf_w-f_w\right)t+j\phi_b\left(t\right)-j\varphi_0} \end{aligned} \tag{7}$$

where $J_n(\cdot)$ is the Bessel function of the first kind of order n.

Compared to wingbeat, the clutter and insect body movement can be considered as stationary or changing slowly. Thus, the received signal from the clutter and insect body can be removed by high-pass filtering. However, it can be found from (7) that the received signal modulated by wing-beating also has the low-frequency component while $n = \pm 1$. After high-pass filtering, (5) can be written as

$$\sigma\left(t\right)e^{j\phi\left(t\right)} = \sigma_{wingbeat}\left(t\right)e^{j\phi_w\left(t\right)} - j\sigma_0\cos\varphi_0 J_1\left(\frac{4\pi A}{\lambda}\right)e^{j\phi_b\left(t\right)} \tag{8}$$

From equation (8), it can be seen that the second item in equation (5) which represents the low-frequency component is removed by high-pass filtering.

With respect to wingbeat frequency extraction, the Fourier transform can be directly applied on (8). The wingbeat frequency can be obtained based on harmonic-pairs positions in signal spectrum, because the wing-beating can make the signal generate a series of harmonic pairs with the frequency interval of wingbeat frequency (See equation (7)).

Besides, the signal phase analysis of wing-beating can be also used for wingbeat frequency extraction. The signal phase of (8) can be expressed as

$$\tilde{\phi}_w\left(t\right) = \tan^{-1}\frac{\sigma_0\sin\left(2\pi f_w t + \varphi_0\right)\sin\left(\phi_b\left(t\right) + \frac{4\pi A}{\lambda}\sin\left(2\pi f_w t\right)\right) - \sigma_0\cos\varphi_0 J_1\left(\frac{4\pi A}{\lambda}\right)\cos\phi_b\left(t\right)}{\sigma_0\sin\left(2\pi f_w t + \varphi_0\right)\cos\left(\phi_b\left(t\right) + \frac{4\pi A}{\lambda}\sin\left(2\pi f_w t\right)\right) + \sigma_0\cos\varphi_0 J_1\left(\frac{4\pi A}{\lambda}\right)\sin\phi_b\left(t\right)} \tag{9}$$

Note that $J_1\left(4\pi A/\lambda\right) < 1$ and moreover, if the starting time of signal processing is properly selected to make sure φ_0 to be the odd multiple of $\pi/2$, the signal intensity of the low-frequency component approximately becomes zero and far smaller than the signal intensity induced by wing-beating. Consequently, the signal phase induced by wing-beating in

(9) can be expressed as

$$\tilde{\phi}_w\left(t\right)=\phi_b\left(t\right)+\frac{4\pi A}{\lambda}\sin\left(2\pi f_w t\right) \tag{10}$$

As thus, Fourier transform can be also used to extract wingbeat frequency in signal phase spectrum.

Note that the effective vibration amplitude A can be derived from the second component (that is the phase modulation by wing-beating) in Eq. (10). By applying a band-pass filter around the frequency of wing-beating, the second component can be extracted and thus the effective vibration amplitude can be analyzed.

Precision analysis of wingbeat frequency measurement

As aforementioned, the clutter and body signal can be removed by high-pass filtering and the low-frequency component in wingbeat signal could be also ignored. Thus, the signal model of wingbeat frequency measurement can be written as

$$x\left(n\right)=\sigma_0\sin\left(2\pi f_w Tn+\varphi_0\right)e^{j\frac{4\pi A}{\lambda}\sin\left(2\pi f_w Tn\right)}+w\left(n\right) \tag{11}$$

where $w\left(n\right)$ is the white Gaussian noise with zero mean. In addition, the Doppler phase of insect body is not introduced into the measurement model, because it only causes frequency shift of the received signal and has no influence on wingbeat frequency measurement.

If only the signal amplitude modulation is acquired, the signal model will be simplified as

$$x\left(n\right)=\sigma_0\sin\left(2\pi f_w Tn+\varphi_0\right)+w\left(n\right) \tag{12}$$

After tedious derivation of Fisher information matrix, the Cramer-Rao lower bounds (CRLB) of amplitude- and phase-based wingbeat frequency estimate can be obtained

$$
\begin{aligned}
Var_A\left(\hat{f}_w\right) &\geq \frac{6\sigma_w^2}{\pi^2\sigma_0^2T^2N\left(N^2-1\right)} \\
Var_P\left(\hat{f}_w\right) &\geq \frac{3\sigma_w^2\lambda^2}{2\pi^2\sigma_0^2T^2N\left(N-1\right)\left(2N-1\right)\left(\lambda^2+12\pi^2A^2\right)}
\end{aligned}
\qquad (13)
$$

where σ_w^2 is the variance of the white Gaussian noise and N is the signal sampling number. It is found that for the same radar parameters, the Cramer-Rao lower bound of amplitude-based wingbeat frequency estimate is about three times than that of phase-based method when the vibration amplitude induced by wing-beating is at sub-millimeter order level.

According to the above two equations, it can be found that wingbeat frequency estimate is influenced by several factors, including radar wavelength, sampling rate, sampling number, the backscattered signal intensity of wings, the effective vibration amplitude of wing-beating and noise level. Generally, the shorter wavelength, longer observation time, stronger backscattered signal, larger vibration amplitude and lower noise level are beneficial for wingbeat frequency estimate for phase-based method and potential to achieve better precision.

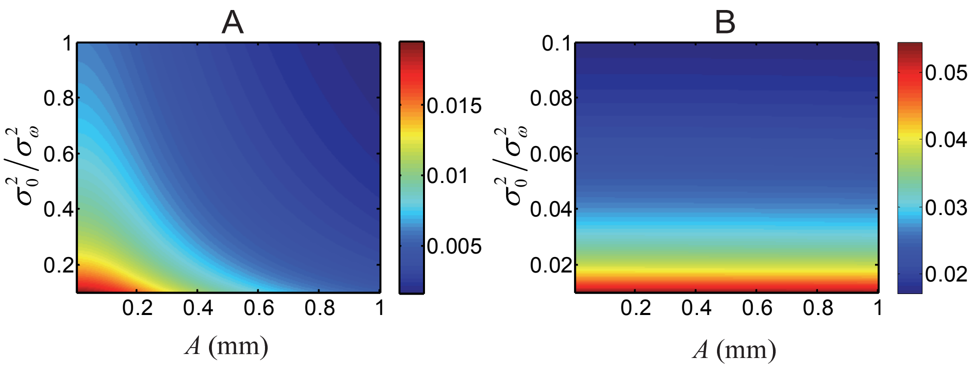

Figure S3. The CRLB simulations of wingbeat frequency estimate accuracy based on (a) W-band radar and (b) S-band radar parameters.

Fig. S3 shows CRLB simulations of wingbeat frequency estimate according to W-band and S-band radar parameters under different backscattered signal intensities and effective vibration amplitudes, where the sampling rate and the sampling number are set to 0.5 ms and 2000 for W-band radar while they are 0.2 ms and 4000 for S-band radar. The backscattered signal intensities are different for experimental insects with body length ranging from about 10 mm-40 mm. Moreover, the scattering mechanism is significantly different that Rayleigh scattering mainly happens to S-band experiments while the scatterings of these insects for W-band locate in resonance or optical region. Based on signal power analysis of high resolution range profiles, the ratio between the received signal power from wing-beating and signal noise power is approximately from 10^{-1} to 1 for W-band radar while this ratio is one order of magnitude smaller for S-band radar in general (see Table 1 in the text). In addition, the effective vibration amplitude can be derived from the wingbeat phase undulation, which is about millimeter order of magnitude or even smaller.

From simulation results, it can be seen that the W-band coherent radar theoretically has better estimate accuracy, which is consistent with our experimental results. However, our wingbeat frequency measurement precision does not achieve CRLB in the experiments, probably due to non-optimal estimate method. Therefore, theoretically, wingbeat frequency measurement based on coherent radar could realize higher precision and thus the optimal estimate method still needs to be investigated further.

Reference

1. Levanon, N. Stepped-frequency pulse-train radar signal, *IEE Proc.-Radar Sonar Navig.*, **149: 6**, 297-309 (2002)
